# A systematic survey in *Arabidopsis thaliana *of transcription factors that modulate circadian parameters

**DOI:** 10.1186/1471-2164-9-182

**Published:** 2008-04-21

**Authors:** Shigeru Hanano, Ralf Stracke, Marc Jakoby, Thomas Merkle, Malgorzata A Domagalska, Bernd Weisshaar, Seth J Davis

**Affiliations:** 1 Max Planck Institute for Plant Breeding Research, Carl-von-Linné-Weg 10, D-50829 Cologne, Germany; 2 Bielefeld University, Department of Biology, Chair of Genome Research, D-33594 Bielefeld, Germany; 3 Research Institute for Biological Sciences OKAYAMA Okayama 716-1241 Japan; 4 Department of Biology, University of York. PO Box 373, York, YO10 5YW; UK

## Abstract

**Background:**

Plant circadian systems regulate various biological processes in harmony with daily environmental changes. In *Arabidopsis thaliana*, the underlying clock mechanism is comprised of multiple integrated transcriptional feedbacks, which collectively lead to global patterns of rhythmic gene expression. The transcriptional networks are essential within the clock itself and in its output pathway.

**Results:**

Here, to expand understanding of transcriptional networks within and associated to the clock, we performed both an *in silico *analysis of transcript rhythmicity of transcription factor genes, and a pilot assessment of functional phenomics on the *MYB*, *bHLH*, and *bZIP *families. In our *in silico *analysis, we defined which members of these families express a circadian waveform of transcript abundance. Up to 20% of these families were over-represented as clock-controlled genes. To detect members that contribute to proper oscillator function, we systematically measured rhythmic growth *via *an imaging system in hundreds of misexpression lines targeting members of the transcription-factor families. Three transcription factors were found that conferred aberrant circadian rhythms when misexpressed: *MYB3R2*, *bHLH69*, and *bHLH92*.

**Conclusion:**

Transcript abundance of many transcription factors in Arabidopsis oscillates in a circadian manner. Further, a developed pipeline assessed phenotypic contribution of a panel of transcriptional regulators in the circadian system.

## Background

The *Arabidopsis thaliana *(Arabidopsis) circadian clock drives growth and development in response to daily and seasonal change [1]. This is of ecological relevance as the clock has been shown to be critical for plant fitness and appears to be evolving in correlation with latitude [2,3]. In Arabidopsis, the clock system is proposed to be composed of integrated transcriptional feedbacks [4-6]. These loops drive global gene expression rhythms [7]. In fact, estimates of the total global consortium of cycling genes has ranged from 2% to 36% of all Arabidopsis transcripts [8-10]. These global regulatory patterns of transcript abundance demonstrate that whole regulatory and metabolic pathways are under clock control [8,10,11]. This exquisitely coordinated regulation is thought to be the purpose of the clock. Overall one can see an emerging, systems-level understanding of the complicated biological mechanisms composed of transcriptional networks driven by the clock. Functional tests of these hypotheses are required to fully expand the integrated network.

Understanding the molecular nature of the circadian oscillator is an ongoing task. Within the currently understood core of the oscillator are the sequence related MYB-like factors *CIRCADIAN CLOCK ASSOCIATED 1 *(*CCA1*) and *LATE ELONGATED HYPOCOTYL *(*LHY*). These genes were first discovered through misexpression studies, as overexpression of either was found to generate an arrhythmic clock [12,13]. Further work on these factors [14], and the identification and characterization of other clock genes [15], resulted in an elegant description of the rhythm-driving oscillator [16-18]. Here a four-loop model has been proposed where in the core of this oscillator lies CCA1/LHY and the pseudo-response regulator *TIMING OF CHLOROPHYLL A/B-BINDING PROTEIN *(*CAB2*, also termed LHCB1*1) *GENE EXPRESSION 1 *(*TOC1*) [16-18]. This core was confirmed as the *cca1 lhy toc1 *triple mutant has seriously attenuated rhythmic behavior [19]. CCA1/LHY are genetically transcriptional repressors of *TOC1*, and TOC1 is a positive genetic factor, with an as of yet unproven biochemical function [20], that functions in transcriptional induction of *CCA1 *and *LHY*. The CCA1/LHY loop is further regulated by a morning loop that contains the *TOC1 *sequence-related genes *PSEUDORESPONSE REGURATOR 9 *(*PRR9*) and *PRR7*. In turn, the TOC1 arm of the clock is also regulated by a loop that includes the *GIGANTEA *(*GI*) flowering-time gene [15,17]. Current models infer as of yet unidentified transcription factors in this looped network [16].

Circadian-regulated transcription factors should confer the complete array of phased rhythms of transcript accumulation that is observed [8,10]. As for example, the MYB-like transcription factors CCA1 and LHY, thought core for normal clock function, are predicted to drive output regulation [10,8]. Additionally, the MYB-transcription factor EARLY PHYTOCHROME RESPONSIVE 1 (EPR1), the MADS-domain factor FLOWERING LOCUS C (FLC), and a GARP transcription factor, LUX ARRYTHMO (LUX), were also reported to be involved in circadian system [21-23]. These three genes could additionally control a suite of transcript outputs from the clock. Another example of the regulation of circadian outputs by clock-controlled transcription factors is the regulation of the anthocyanin biosynthesis pathway, where structural enzymes for this secondary metabolite are encoded by genes coordinately regulated by a cycling output transcription factor called as PRODUCTION OF ANTHOCYANIN PIGMENT 1, PAP1 [10]. Thus, not all rhythmic transcription factors feedback to the oscillator. We believe it is likely that a small set of transcription factors await to be discovered that can modulate clock function, and just as importantly, we expect that a large set of transcription factors are themselves regulated at the transcript accumulation level to drive the physiological suite of rhythmic outputs.

For the circadian clock to drive rhythmic expression of such a large part of the genome, and for these genes to be phased at all times of the subjective day (no phase bias exists, as shown by [10]), a suite of transcription factors must be implicated in the clock-output system. The Arabidopsis genome encodes more than 1500 transcription factors that belong to more than 30 different families [24]. Each family of transcription factors was characterized based on the definition of containing a highly conserved DNA-binding domain(s). For example, the Arabidopsis genome contains 133 members of MYB transcription factor superfamily, 162 genes encoding basic helix-loop-helix (bHLH) transcription factors, 75 distinct members of basic region/leucine zipper motif (bZIP) transcription factors [25-27]. Some transcription factors were reported as activators and repressors to compose complexly integrated regulatory loops in the plant circadian system [17]. However, functional characterization of the vast majority of Arabidopsis transcription factors still remains.

Here, we took two overlapping genomic approaches to further catalog the repertoire of transcription factor use within the oscillator and in expression of output traits. These companion approaches identify previously uncharacterized plant genes involved in the circadian system, and further dissects this complex signaling network. For this, we surveyed existing microarray results of the *MYB*, *bHLH*, and *bZIP *transcription factor families and determined those that are clock regulated, and separately, systematically analyzed circadian rhythms in misexpression mutants targeting transcription factors, *via *time-lapse imaging. We report the discovery of three misexpression lines that have altered circadian parameters. Our suite of analyses lead us to conclude that although many transcription factors do not contribute to normal clock function, transcription factors previously non-described within the clock can be discovered through systematic tests, including computational surveys.

## Results

### Defining circadian expression within transcription-factor families

As suites of transcripts are clock regulated in Arabidopsis [10], we hypothesized that this was due to rhythmic accumulation of transcription factors. MYB, bHLH, and bZIP are the predominant factor families previously implicated in light- and clock-regulated accumulation of targets [25-27], so we decided to test how prevalent individual rhythms are within these families. Previously, we have collectively reported no less than 368 genes predicted to encode transcription factors in the MYB (131), bHLH (162), and bZIP (75) transcription factor families in Arabidopsis [25-27]; these were the target pools queried. We accessed expression profiles of circadian experiments, NASCArrays Experiment Reference Number: NASCARRAYS-108 [28], using Affymetrix ATH1 arrays containing 22,746 probe sets in the public microarray database, GENEVESTIGATOR [28,29]. This probe set represents 122 *MYB*, 111 *bHLH*, and 67 *bZIP *genes. In these circadian datasets, a total of 185 of these genes (51 *MYB*, 81 *bHLH*, and 53 *bZIP*) were found to be expressed at least one time point (at p < 0.06). We noticed that the expression of more than half of *MYB *genes was below detection level on these hybridization samples. Perhaps this implies that many *MYB *transcripts in this experimental protocol were tissue or growth-stage specific. In contrast, most *bHLH *and *bZIP *genes were detected in this array experiment. The expression data we processed from these 185 genes was sufficient for further *in silico *analyses.

**Figure 1:**
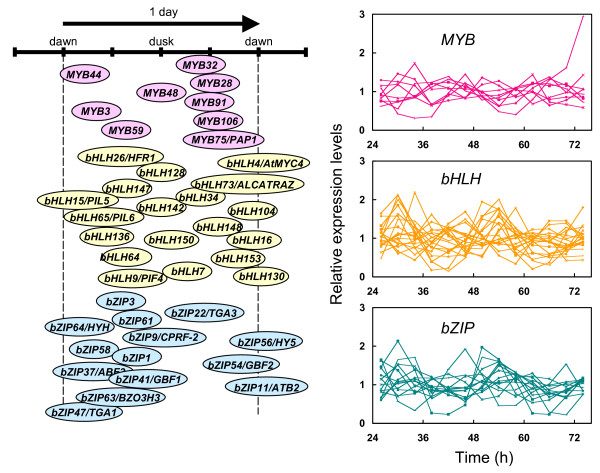
**Oscillation peaks of various transcription factors**. Transcripts from 42 transcription factors oscillate with certain peaks in circadian phase of one subjective diurnal day. The peak expression of these genes is illustrated (*Z*T h). MYB, bHLH, and bZIP transcription factors are colored in pink, yellow, and blue, respectively. Each trace illustrating the cycling patterns of expression is shown on the right side of this figure.

We scored the expression values of the 185 genes using the modified Cosinor analysis [30]. This analysis was used successfully in previous experiments to score the circadian expression for genes in Drosophila, mouse, and Arabidopsis [10,31,28]. A previous study using this approach in Arabidopsis employed three threshold scales of significance to assess probability (pMMC-β): < 0.02, 0.05, and 0.1 [28]. We used the same confidence cut-offs to define rhythmic genes (Table 1). A total of 42 transcription factor genes with a pMMC-β value of 0.05, which reflected 9 MYB, 19 bHLH, and 14 bZIP transcription factors, were scored as rhythmic (Table 1, Additional file 1). The percentages of rhythmic *bHLH *and *bZIP *genes within each respective family were similar to, or even slightly higher than, that of the set of "all" genes (Table 1). The percentage of rhythmic *MYB *genes was less than that of the *bHLH*, *bZIP*, and the set of all genes; however, noteworthy is the percentage of expressed-*MYB *on the array that was, itself, lower than other sets. A graphic representation of the expression patterns illustrates when a given peak occurred during transcription factor oscillation (in (h) hours relative to *zeitgeber *time, which is the time of the last external temporal cue such as the dawn signal of lights-on), (Figure 1). It was noted that many *bZIP *genes were transcribed during the photophase of the day, whereas many *MYB *genes peaked during the skotophase (Figure 1). These collective results highlight that, as expected, many transcription factors oscillate, and do so at many discreet phases of the daily cycle.

**Table 1:** *MYB*, *bHLH *and *bZIP *genes scored as rhythmic by COSOPT. Total number of *MYB*, *bHLH *and *bZIP *on the genome, array and expressed in the circadian experiment (p < 0.1) is shown. Number of rhythmic genes is represented as total and percentage of the transcription factors on the array. The rhythmic scoring was performed at three different pMMC-beta thresholds.

**Genome**	**GENEVESTIGATOR**			**COSOPT (pMMC-beta)**			**Rhythmic (%) on the array**		
			
	T**otal**	**All**	**(p < 0.1)**	**< 0.02**	**< 0.05**	**< 0.10**	**< 0.02**	**< 0.05**	**<0.10**
MYB	131	122	51	4	9	16	3.3	7.4	13.1
*bHLH*	162	111	81	12	19	27	10.8	17.1	24.3
*bZIP*	75	67	53	6	14	17	9.0	20.9	25.4
Total	368	300	185	22	42	60	7.3	14.0	20.0
						(all genes in Edwards K. D. et.al.)			
							7.6	15.4	22.54

### Circadian function of transcription factors

In the *in silico *analysis (Figure 1), we found several night-expressed transcription factors. We wondered if such transcription factors could be the as of yet unidentified clock components proposed by current models [16,17]. To generically test this hypothesis, we surveyed T-DNA tagged lines targeting these transcription factors, obtained from the public stock center, and measured circadian rhythms of these mutants in our system. However, no strong alternations of the circadian rhythm were observed in any of these lines under our assay condition (data not shown). Our observation suggested that none of these factors are the predicted clock element, and thus alternative approaches must be used to define such circadian mutants.

To identify transcription factors that function within the circadian system, as part of control or slave oscillator, we assessed rhythmic output of lines targeted to misexpress a given transcription factor. These experiments, because they are overexpression studies, allowed us to circumvent genetic redundancy. We feel that this is a particularly important consideration in the Arabidopsis clock, as the Mybrelated sequences CCA1 and LHY have strong circadian defects when overexpressed [12,13], but have only mild phenotypes as single gene loss-of-function alleles because of the redundancy inherent in the system, [14]. Our hypothesis was that members of the *MYB*, *bHLH*, and *bZIP *families are as of yet uncharacterized components of the oscillator and/or are slave components. As a test of this hypothesis, we made use of 198 plants over-expressing 39 *MYB *genes, 29 *bHLH *genes, and 4 *bZIP *genes (Table 2, Additional file 2). Overexpression of each transcription factor was confirmed by RT-PCR on rosette leaf cDNA from T1 plants (data not shown, *e.g*. Additional file 5).

**Table 2:** Plants targeting transcription factors analyzed in this study. A total of 198 mutants, including 198 misexpression were analyzed in this study. The misexpression lines targeted 39 *MYB*, 29 *bHLH *and 4 *bZIP *genes. The period phenotypes of 19 out of 22 over-expression lines were not consistent in multiple transgenics. Aberrant clock precision was only observed in 1 out of 3 bZIP48-ox lines. Numbers in ( ) indicated lines over-expressing same genes, which support that the phenotypes were caused by misexpression of the genes.

		**Lines**	**Genes**	**Period**	**Phase**	**Precision**
MYB	over expression	108	39	11(0)	5(5)	0
bHLH	over-expression	82	29	11(3)	5(5)	0
bZIP	over-expression	8	4	0	0	1(0)

Total	lines	198		22	10	1
Total	genes		72	1	2	0

**Figure 2:**
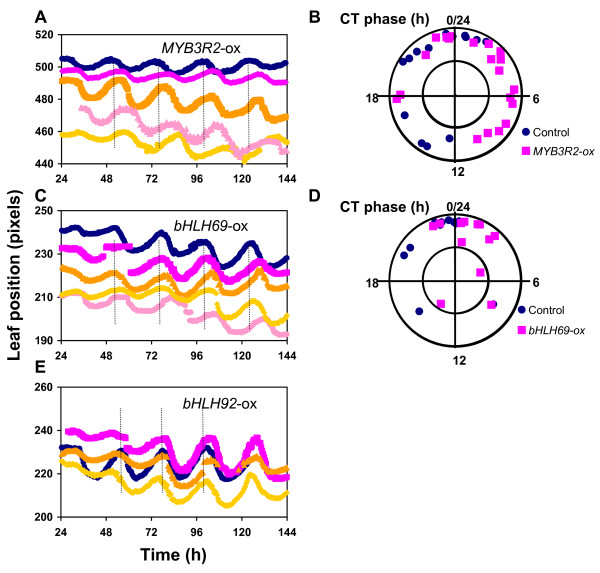
**Representative leaf movement data for lines expressing a clock phenotype**. Leaf movement rhythms were assayed under constant light for approx. 1 week (n = 14–28). (**A**, **C**, **E**) Representative traces of rhythmic leaf movement of wild-type (blue circles) and ox-lines (other colored symbols) are shown. (**B**, **D**) The phase angles normalized to a 24-h cycle (CT phase) are plotted with relative amplitude errors (RAE), which indicate the robustness of the rhythm (the lower the RAE the more robust the rhythm). The center of the circle represents a high RAE (= 1). (**A**, **B**) *MYB3R2*-ox, (**C**, **D**) *bHLH69*, and (**E**) *bHLH92*.

We constructed a high-throughput time-lapse imaging system, similar to one previously reported [32]. With this system, we measured the circadian rhythms of leaf movement in each transgenic line. For this, the seedlings were entrained under 24-hour light-dark cycles for ~10 days, and then the leaf positions of individual plants were imaged under constant light (LL) for an additional 7 days. The circadian parameters of the change in leaf position were analyzed (Additional files 2, 3, 4, 5 &6). We found 33 misexpressors with circadian phenotypes, and 13 out of 33 misexpressor lines targeted the three genes *MYB3R2*, *bHLH69*, and *bHLH92 *(Figure 2; Tables 2 and 3). *MYB3R2*-ox and *bHLH69*-ox displayed a 4–8 hour delayed phase of leaf movement rhythms (Figure 2A and 2C). Statistical analysis indicated no significant differences in circadian periodicity (Table 3; Additional files 2 and 3), while the phase difference was significant (Figure 2A–D). *bHLH92*-ox plants exhibited a 0.5~2 hour lengthened periodicity phenotype, compared to the wild type (Figure 2E). The periodicity differences were statistically significant; WT, 24.16 ± 0.45; *bHLH92*-ox line A, 26.32 ± 0.77; line B, 25.09 ± 0.32; line D, 25.16 ± 0.36; line E, 24.76 ± 0.41 (p < 0.05, R. A. E. < 0.4) (Table 3). Thus, MYB3R2 and bHLH69 controls circadian phase, and bHLH92 contributes to the regulation of circadian periodicity.

**Table 3:** Misexpression lines exhibit aberrant clock phenotype. Mean circadian periods of leaf movement in Arabidopsis plants misexpressing transcription factors and control seedlings, estimated with BRASS. S.E.M.: standard error of the mean, n: number of contributing leaf traces.

**Genes**	**Line name**	**n**	**Period (±) S.E.M**	**Phenotype**
**MYB3R2 (At4g00540)**	**1**	43	24.44 ± 0.46	phase
	**2**	28	24.53 ± 0.29	phase
	**5**	12	24.00 ± 0.19	phase
	**6**	11	26.69 ± 0.28	Phase/long
	**7**	12	25.06 ± 0.33	phase
**Control**		18	24.65 ± 0.27	
				
**bHLH69 (At4g30980)**	**A**	47	25.20 ± 0.44	phase
	**B**	51	25.39 ± 0.33	phase
	**D**	17	24.92 ± 0.21	phase
	**E**	8	24.70 ± 0.37	phase
	**I**	22	24.19 ± 0.25	phase
**Control**		27	24.94 ± 0.27	
				
**bHLH92 (At5g43650)**	**A**	27	26.32 ± 0.77	long
	**B**	18	25.09 ± 0.32	long
	**D**	24	25.16 ± 0.36	long
	**E**	24	24.76 ± 0.41	long
**Control**		18	23.76 ± 0.34	

During imaging experiments, we identified additional phenotypically altered lines in the pools of misexpression transgenics (Table 2, Additional files 2, 3 &4). A total of 19 over-expression lines exhibited aberrant periodicity, and one of the *bZIP*ox plants (*bZIP48*-ox) lacked clock precision. However, phenotypes from these lines were not confirmed by other lines targeting the over-expression of the same gene. One plausible explanation is that these detected phenotypes are not correlated to the targeted gene, and perhaps was caused by coincident mutations occurring during TDNA transformation.

### *MYB3R2*, *bHLH69*, and *bHLH92 *could influence circadian rhythms

**Figure 3:**
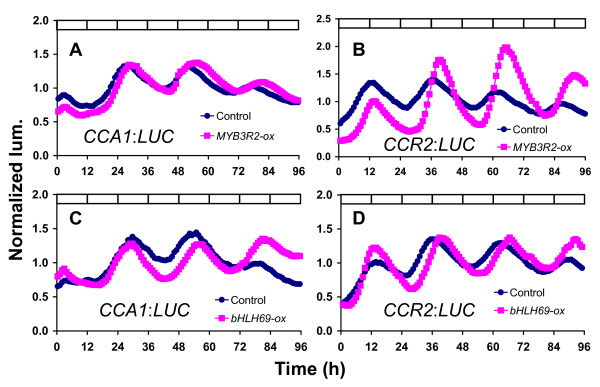
**Confirmation of transcriptional clock phenotypes under constant light**. Seedlings harboring *CCA1*:*LUC *or *CCR2*:*LUC *reporter genes were monitored under constant light for 4–5 days. Representative traces of rhythmic expression of ox-plants (pink squares) and wild-type (blue circles) are shown. (**A**, **B**) *MYB3R2*-ox, (**C**, **D**) *bHLH69*-ox. (**A**, **C**) *CCA1*:*LUC*, (**B**, **D**) *CCR2*:*LUC*.

To confirm the clock phenotypes observed in *MYB3R2*, *bHLH69*, and *bHLH92 *over-expression lines, we employed the promoter:*LUCIFERASE *(*LUC*) system as an assay that here is used to detect rhythmic patterns of gene expression [33]. *LUC *fusions to the well-characterized circadian-regulated promoter, *CCA1 *and to the *COLD-AND CIRCADIAN-REGULATED 2 *(*CCR2*, also termed *AtGRP7*) promoter were separately introduced into these *MYB3R2*-ox, *bHLH69*-ox and *bHLH92*-ox plants *via *fertilization. If these transcription factors act upstream of the clock, both *CCA1 *and *CCR2 *oscillations would be altered. Alternatively, if these genes act downstream, controlling the output pathway that regulates leaf movement, the *CCA1 *rhythm intimately associated with clock function would not be affected. *MYB3R2*-ox exhibited delayed phase phenotypes of both *CCA1 *and *CCR2 *rhythms under LL (Figure 3A and 3B). *bHLH69*-ox also delayed the phase of the *CCA1 *and *CCR2 *rhythm (Figure 3C and 3D). In our mathematical analysis, we could not find any significant effects on the circadian periodicity (Additional file 7A). The second peak positions of *CCA1 *rhythms in control, *MYB3R2*-ox, *bHLH69*-ox and *bHLH92*-ox were 51.44 ± 0.44, 54.22 ± 0.71, 53.18 ± 0.45 and 53.49 ± 1.03, respectively (Additional file 7B). P-values for *MYB3R2*-ox and *bHLH69*-ox were less than 0.01, while p-value for bHLH92-ox was 0.07. In the second peaks of CCR2 rhythm, the values in control, MYB3R2-ox and bHLH69-ox were 37.01 ± 0.23, 39.37 ± 0.63 and 39.81 ± 0.89 (p < 0.01). Thus, we found that overexpression of *MYB3R2 *and *bHLH69 *altered both *CCA1 *and *CCR2 *rhythms, suggesting that these genes can control core-clock functions, rather than being specific to the leaf-movement-output pathway.

**Figure 4:**
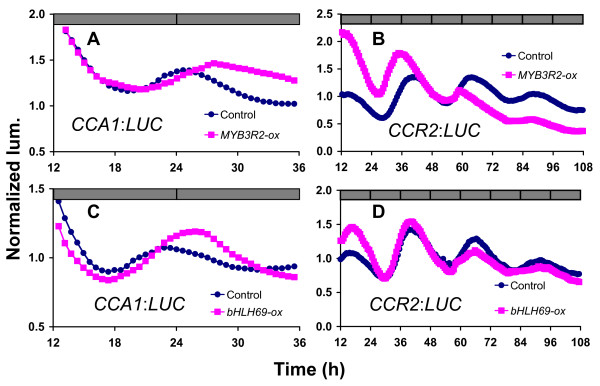
**Confirmation of transcriptional clock phenotypes in constant dark**. Seedlings harboring *CCA1*:*LUC *or *CCR2*:*LUC *reporter genes were monitored in constant darkness for 4–5 days. Representative traces of rhythmic expression of oxplants (pink squares) and wild-type (blue circles) are shown. (**A**, **B**) *MYB3R2*-ox, (**C**, **D**) *bHLH69*-ox. (**A**, **C**) *CCA1*:*LUC*, (**B**, **D**) *CCR2*:*LUC*.

We measured *CCA1 *and *CCR2 *rhythms in *MYB3R2*-ox, *bHLH69*-ox, and *bHLH92*-ox plants in constant darkness (DD) (Figure 4). This allows us to compare their behavior to the LL phenotypes. Interestingly, *MYB3R2*-ox plants had an advanced phased *CCR2 *rhythm and a delayed phase of *CCA1 *expression in DD (Figure 4A and 4B). The peak positions in control and *MYB3R2*-ox were 37.44 ± 0.44 and 35.86 ± 0.46 in the *CCR2 *rhythm, and 51.23 ± 0.61 and 53.92 ± 0.69 in the CCA1 rhythm (p < 0.01) (Additional file 7D). A delayed phase of *CCA1 *in DD was seen in *bHLH69*-ox lines (Control = 51.23 ± 0.61, bHLH69-ox = 54.05 ± 0.34; p < 0.01) (Figure 4C and 4D, Additional file 7D). *bHLH92*-ox plants also exhibited a clock phenotype in DD. Here an effect on *CCA1 *phase was detected (Additional file 6B). The values were 53.94 ± 0.57 (p < 0.01). We thus concluded that misexpression of any of these three transcription factors could alter clock parameters. However, the specific nature of the phenotypic effects depended on the light conditions and the output measured.

### *MYB3R2 *and *bHLH69 *could alter clock-gene expression

**Figure 5:**
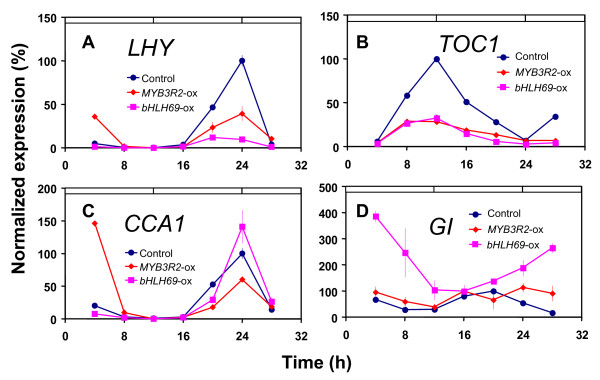
**Clock-gene expression profiles in selected transcription factor misexpression lines**. Seedlings from ox- and wild-type plants were harvested every 4 hours. Total RNA was the substrate for RT-PCR against the coding regions of the core-clock genes *CCA1*, *LHY*, *GI*, or *TOC1*, and as a control, *TUBULIN *(*TUB*). Results are presented as proportional to the average value after normalization with respect to *TUB*. Expression profiles in the control, *MYB3R2*-ox and bHLH69-ox were represented as blue, orange, and pink lines, respectively. (**A**) *LHY*, (**B**) TOC1, (**C**) *CCA1*, and (**D**) *GI*.

As described above, we detected circadian alternations when misexpressing given transcription factors. Because the effect of *bHLH92*-ox was dependent on the light condition, we continued our focus on *MYB3R2*-ox and *bHLH69*-ox to further characterize the molecular basis for their phenotypes. To this end, we analyzed transcripts of the central oscillator genes *CCA1*, *LHY*, *TOC1*, and *GI *in *MYB3R2*-ox and *bHLH69*-ox (Figure 5). The *MYB3R2*-ox and *bHLH69*-ox plants were entrained under 12 hour light/12 hour dark cycles, and then transferred to constant light conditions. Replicate samples from these plants were harvested every 4 hours for RNA isolation and expression analysis using reverse transcriptase (RT)-PCR. Both *MYB3R2*-ox and *bHLH69*-ox were found to result in a repressed transcript level of *LHY *and *TOC1 *(Figures 5A and 5B). *CCA1 *mRNA was found to be slightly decreased in *MYB3R2*-ox, while this was increased in *bHLH69*-ox (Figure 5C). Interestingly, in *bHLH69*-ox, induction of *GI *expression was found to display a nearly opposite phase with high expression level (Figure 5D). At *zeitgeber *time (*z*t) = 4, *LHY *and *CCA1 *mRNA are highly accumulated in *MYB3R2*-ox. This response could be accounted for by an acute light response. Alternatively, a phase delay or defective entrainment in *MYB3R2*-ox might cause the high expression seen at this time-point. Interestingly, the alteration in *GI *expression did not result in a dramatic alteration in the timing of the floral induction (Additional file 8). We suggested that MYB3R2 functions as a regulator of *CCA1*, *LHY*, or *TOC1 *transcription, and suggest that bHLH69 plays a similarly important role to regulate *CCA1 *and *GI *expression.

**Figure 6:**
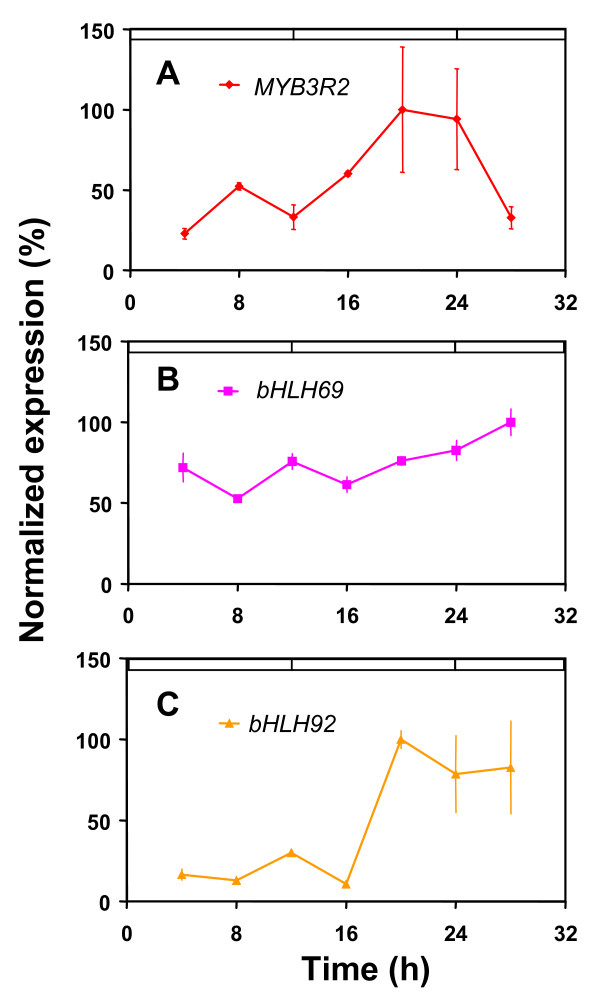
**Transcript accumulation of transcription factors that have clockcontrolling properties**. Replicate seedlings from wild-type plants maintained under constant light were harvested every 4 hours. Total RNA was the substrate for RT-PCR of the coding regions of the transcription factors *MYB3R2*, and *bHLH69 *and *bHLH92*. Results are presented as proportional to the average value after normalization with respect to *TUB*. (**A**) *MYB3R2*, (**B**) *bHLH69*, and (**C**) *bHLH92*.

We next investigated whether the *MYB3R2*, *bHLH69*, and *bHLH92 *genes were transcribed in a circadian manner. We performed this experiment as the expression profiles of these three genes were not part of the publicly available datasets described above [29]. The mRNA accumulation patterns of these transcription factors were assayed by RT-PCR from RNA extracted from plants grown under light-dark cycles and then transferred to LL (Figure 6). *MYB3R2 *and *bHLH92 *were likely to be expressed in a circadian manner with a peak between late night and dawn. In contrast, the mRNA accumulation of *bHLH69 *was not found to oscillate.

## Discussion

### Identification of previously functionally uncharacterized transcription factors in the clock

Here we describe that MYB3R2, bHLH69, and bHLH92 can contribute to the plant-circadian system. Alongside the characterized transcription factors *CCA1*, *LHY*, *EPR1*, *FLC*, and *LUX*, this now adds to the list of transcriptional clock-modulators. We provide evidence that MYB3R2 and bHLH69 influences circadian phase, whereas bHLH92 influences phase and periodicity, dependent on environmental conditions. These effects were not as strong as misexpression of *CCA1*, *LHY*, or *LUX*. It is still unclear how MYB3R2, bHLH69, and bHLH92 function in the clock system. Their specific effects depended on the light environment and on the output measured. Expression analysis showed that misexpression of *MYB3R2 *and *bHLH69 *resulted in altered transcript levels of clock genes; however, the circadian rhythms still kept 24-h periodicity albeit with aberrant phase. Thus, transcription factors identified here may play a role in environmental input to the clock or the mediation of its effects, rather than functioning as central-clock components. Such processes are described [34]. A future effort to explore a detailed analysis of these transcription factors and the identification of target DNA elements remains to be carried out.

Plant circadian systems possess interlocked feedback loops [17-19]. In addition, there are various junctures for signal convergence and divergence in the input and output pathways [35,36]. Though a set of clock regulating transcription factors has already been identified sufficient to describe much of the oscillator framework [11], many other components are believed to still be lacking [10].

We described an additional 20 misexpression lines that exhibit altered clock phenotypes. Whether these genes are components of input core, or output pathways, is as of yet unknown. This was as the phenotypes detected in these lines were not substantiated with alternative transgenic inserts. Forward-genetic analysis of loss-of-function phenotypes of these lines is worth further attention to determine their function in the clock.

A current mathematical model proposes the existence of an unknown transcription factor "*X*", which activates *CCA1 *and *LHY *[16,17]. We tested hundreds of transcription factors as a pilot study of functional phenomics within the circadian clock, but it appears that in this test, we did not find "factor *X*." Still, functional analysis of more than a thousand transcription factors still remains. Thus, our pilot efforts substantiate that it is worthwhile to interrogate, *via *further functional genomic efforts, all known Arabidopsis transcription factors and to analyze their circadian responses.

### Transcriptional networks in clock output pathways

To dissect the transcriptional regulation in circadian clock, we employed a systematic analysis of global-gene expression. In our *in silico *analysis, up to 20% of the transcription factors assayed were clock controlled (Table 1). This percentage is slightly higher than that of "all" circadian rhythmic genes in the Arabidopsis genome [10]. This implies that the circadian oscillations in the output pathways are not just regulated by a select group of transcriptional feedbacks, and actually require a large number of rhythmic transcription factors. In contrast, the mammalian system is reported to only use around 16 cycling transcription factors, which oscillate in a circadian manner, to regulate the clock [37]. The evolution of transcription-factor function and recruitment of molecular targets leads to the linking of many processes of plant physiology to the circadian system. Some components of these systems direct the clock itself, whereas others are only components of rhythmic physiological outputs.

## Conclusion

Here we employed two genomic approaches as a pilot study to explore clock function. We found three genes that could modulate circadian parameters. Many other transcription factors oscillated in a circadian manner. This implicates these factors in physiological circadian outputs. Further exploration, with our described approaches, should bring novel insights in circadian input and output pathways, and identify genes previously non-described as functional within the central-clock oscillator.

## Methods

### Expression analyses through the public database

Gene codes of all transcription factors studied, referred to as AGI numbers, were collected within the Arabidopsis Information Resource. The gene-expression profiles in circadian experiment were available from the public microarray database Genevestigator [29,28]. Expression values from the data were subject to score circadian rhythms with COSOPT [30]. Data was collated without non-linear regression (Additional file 1).

### Generation of transcription factor OX-lines

Full-length coding sequences (ATG-to-Stop) from MYB-, bHLH- and bZIP transcription factors were amplified from respective cDNAs by PCR using attB-sites containing gene-specific primers. Gateway Entry clones were generated *via *BP-reaction using the vector pDONR201 (Gateway system, Invitrogen, USA). *Via *LR-reaction the transcription factor cDNAs were transferred behind the double enhancer cauliflower mosaic virus 35S promoter in the plant expression vector pLEELA [38]. Transgenic lines were generated by *Agrobacterium tumefaciens*-mediated transformation of Arabidopsis plants (Col-0) according to the floral-dip protocol [39]. All transgenic lines were selected and self-fertilized. T2 plants were analyzed in this study.

### Plant growth conditions

Seeds were surface sterilized with a 70% ethanol rinse, immediately followed by a rinse with 33% bleach, and then a twice washed with sterile water. The seeds were then aseptically sown on Murashige-Skoog (MS) 1.5% agar medium containing 3% sucrose (pH 5.7) with suitable antibiotic (25 mg/L Kanamycin or 12 mg/L Phosphinotrycin; dependent on the transgene) followed by stratification at 4°C for 4–5 days. Seedlings were grown for 9 days at 22°C under 12 hr light/12 hr dark cycles of 75 μmol m^-2 ^sec^-1 ^cool white fluorescent light. For flowering-time measurements, experiments were as described [40].

### Leaf movement measurement

After 9 days of entraining growth, seedlings were transferred to fresh MS 1.5% agar medium (pH 5.7) containing 3% sucrose without antibiotic, and then agar blocks harboring single seedlings were placed to 25-well square tissue culture dishes (Bibby Sterilin). A set of twenty seedlings in a set within each dish were viewed from the side from plates in a vertically placement. The seedlings were entrained as described above for another day, and then were placed into a growth chamber for imaging over 1 week under constant white light (25–40 μmol m^-2 ^sec^-1^) at a constant 22°C. A total of 14 dishes, containing 280 seedlings, were prepared and imaged with 14 monochromatic charge coupled device video cameras per an experiment. Images of seedlings from every camera were transferred to a computer *via *a Flashbus card and through a custom-built parallel-port controller unit (Universal Imaging, Germany) (system development by Visitron Systems), and were captured and saved every 30-min with a computer program Metaview 4.5 (Universal Imaging) over a week. The vertical positions of primary leaves from the images were measured and analyzed using Metamorph and BRASS, the latter provided by Prof. Andrew Millar (University Edinburgh) as described [28,32]. Period lengths were estimated from the leaf movement data by the fast Fourier transform nonlinear least-squares method [41]. Mean period estimates for each line were based on 10–20 leaf traces from two to four independent experiments analyzed.

### Luciferase imaging

Imaging was performed as described *via *established protocols, where the light was provided from red and blue light-emitting diodes at ~2 μmol m-2 s-1 [5,42,43]. Period length and Relative Amplitude of error (RAE) were estimated using FFT-NLLS program [41].

### RNA isolation and reverse transcriptase-PCR

Seedlings grown for 1 week under LD cycles and replicate samples were harvested every 4 hours under LL conditions. Total RNA was isolated from the seedlings using the RNeasy Plant Mini Kit (Qiagen, USA), and then was treated with DNase I before reverse transcription. Reverse transcription was performed on 1.0 μg of total RNA with SuperscriptII (Invitrogen). Quantitative PCR were performed with iQ5 real-time PCR system (BIO-RAD). Gene-specific primers were described previously: *CCA1*, *LHY *and *TOC1 *[44], *GI *and *TUB *[15]. Primers for *MYB3R2*, *bHLH69 *and *bHLH92 *were designed as follow:

*MYB3R2*-FW, 5'-CTTGGACCACAGAGGAAGAAGT-3'

*MYB3R2*-RV, 5'-TGTTGTTGGTGGTGGTAACCTA-3'

*bHLH69*-FW, 5'-CCATCCTAATGACGCTCTCTTC-3'

*bHLH69*-RV, 5'-ATCAGTGGCTTGACCTCTCCTA-3'

*bHLH92*-FW, 5'-CTGAGAAAGAATTGGGAGGAGA-3'

*bHLH92*-RV, 5'-GACCATCCTTTGCTGATTTTTC-3'

## Authors' contributions

SH, RS, MJ, BW, and SJD conceived the experiments. SH, MAD, and SJD wrote the paper. RS, MJ, TM, and BW generated and confirmed overexpression and insertion lines. SH carried out the circadian experiments. SH and MAD carried out the *in silico *analyses.

## Supplementary Material

Additional file 1 **Supplemental Table 1 – Circadian regulated *MYB*, *bHLH *and *bZIP *genes**. Circadian expression values available in the public database GENEVESTIGATOR were scored for circadian regulation using the modified cosinor analysis program COSOPT. Mean of expression levels, period length, phase values (ZT) and pMMC-β are represented. COSOPT (pMMC-β < 0.05) without linear regression are listed here. Click here for file

Additional file 2 **Supplemental Table 2 – Estimated period length of transgenic lines overexpressing MYB transcription factors**. Mean circadian periods of leaf movement in Arabidopsis plants misexpressing transcription factors and control seedlings, estimated with BRASS. S.E.M.: standard error of the mean, n: number of contributing leaf traces. Click here for file

Additional file 3 **Supplemental Table 3 – Estimated period length of transgenic lines overexpressing bHLH transcription factors**. Mean circadian periods of leaf movement in Arabidopsis plants misexpressing transcription factors and control seedlings, estimated with BRASS. S.E.M.: standard error of the mean, n: number of contributing leaf traces. Click here for file

Additional file 4 **Supplemental Table 4 – Estimated period length of transgenic lines overexpressing bZIP transcription factors**. Mean circadian periods of leaf movement in Arabidopsis plants misexpressing transcription factors and control seedlings, estimated with BRASS. S.E.M.: standard error of the mean, n: number of contributing leaf traces. Click here for file

Additional file 5 **Figure S1 – Confirmation of over-expression of *MYB3R2 *and *bHLH69***. Replicate seedlings from wild-type plants maintained under constant light were harvested every 4 hours. Total RNA was the substrate for RT-PCR of the coding regions of the transcription factors *MYB3R2*, *bHLH69 *and *bHLH92*. Results are presented as proportional to the average value after normalization with respect to *TUB*. (**A**) *MYB3R2 *and (**B**) *bHLH69*. Click here for file

Additional file 6 **Figure S2 – Confirmation of transcriptional clock phenotype of *bHLH92*-ox in constant light and in constant dark**. Seedlings harboring *CCA1*:*LUC *reporter genes were monitored for 4–5 days both under LL (**A**) or in DD (**B**). Representative traces of rhythmic expression of ox-plants (pink squares) and wild-type (blue circles) are shown. (**C**) Relative Amplitude Error (R. A. E.) calculated from the data under LL was plotted against period (h). bHLH92-ox exhibited a slightly long periodicity phenotype. Click here for file

Additional file 7 **Figure S3 – Estimated period and phase of *MYB3R2*-ox, *bHLH69*-ox and *bHLH92*-ox**. Estimated period length and phase values were calculated by BRASS. (**A**) Estimated period of *CCA1 *rhythm. (**B**) Peak positions of second peak in *CCA1 *rhythm. (**C**) Estimated period of CCR2 rhythm. (**D**) Peak positions of second peak in *CCR2 *rhythm. Data are presented as mean ± S.E. with *n *of 12–24 plants. * P = 0.01. No significant difference in periodicity was observed (**A **and **C**). Click here for file

Additional file 8 **Figure S4 – The effects of over-expression of *MYB3R2 *and *bHLH69***. Flowering time of *MYB3R2*-ox and *bHLH69*-ox plants was measured under long day. Leaf number at flowering time were plotted against the genotype and line tested. Data are presented as mean ± S.E. with *n *of 9–14 plants. * P = 0.038. No significant differences were detected in the flowering time of other lines. Click here for file

## References

[B1] McClung CR (2006). Plant circadian rhythms. Plant Cell.

[B2] Michael TP, Salome PA, Yu HJ, Spencer TR, Sharp EL, McPeek MA, Alonso JM, Ecker JR, McClung CR (2003). Enhanced fitness conferred by naturally occurring variation in the circadian clock. Science.

[B3] Dodd AN, Salathia N, Hall A, Kevei E, Toth R, Nagy F, Hibberd JM, Millar AJ, Webb AA (2005). Plant circadian clocks increase photosynthesis, growth, survival, and competitive advantage. Science.

[B4] McWatters HG, Kolmos E, Hall A, Doyle MR, Amasino RM, Gyula P, Nagy F, Millar AJ, Davis SJ (2007). ELF4 is required for oscillatory properties of the circadian clock. Plant Physiol.

[B5] Ding Z, Millar AJ, Davis AM, Davis SJ (2007). TIC encodes a nuclear regulator in the Arabidopsis thaliana circadian clock. Plant Cell.

[B6] Kolmos E, Davis SJ (2007). ELF4 as a central gene in the circadian clock. Plant Signaling and Behavior.

[B7] Michael TP, McClung CR (2003). Enhancer trapping reveals widespread circadian clock transcriptional control in Arabidopsis. Plant Physiol.

[B8] Davis SJ, Millar AJ (2001). Watching the hands of the Arabidopsis biological clock. Genome Biol.

[B9] Schaffer R, Landgraf J, Accerbi M, Simon V, Larson M, Wisman E (2001). Microarray analysis of diurnal and circadian-regulated genes in Arabidopsis. Plant Cell.

[B10] Harmer SL, Hogenesch JB, Straume M, Chang HS, Han B, Zhu T, Wang X, Kreps JA, Kay SA (2000). Orchestrated transcription of key pathways in Arabidopsis by the circadian clock. Science.

[B11] Hanano S, Davis SJ (2007). Mind the clock. Plant Signaling and Behavior.

[B12] Schaffer R, Ramsay N, Samach A, Corden S, Putterill J, Carre IA, Coupland G (1998). The late elongated hypocotyl mutation of Arabidopsis disrupts circadian rhythms and the photoperiodic control of flowering. Cell.

[B13] Wang ZY, Tobin EM (1998). Constitutive expression of the CIRCADIAN CLOCK ASSOCIATED 1 (CCA1) gene disrupts circadian rhythms and suppresses its own expression. Cell.

[B14] Mizoguchi T, Wheatley K, Hanzawa Y, Wright L, Mizoguchi M, Song HR, Carre IA, Coupland G (2002). LHY and CCA1 are partially redundant genes required to maintain circadian rhythms in Arabidopsis. Dev Cell.

[B15] Mizoguchi T, Wright L, Fujiwara S, Cremer F, Lee K, Onouchi H, Mouradov A, Fowler S, Kamada H, Putterill J (2005). Distinct roles of GIGANTEA in promoting flowering and regulating circadian rhythms in Arabidopsis. Plant Cell.

[B16] Locke JC, Millar AJ, Turner MS (2005). Modelling genetic networks with noisy and varied experimental data: the circadian clock in Arabidopsis thaliana. J Theor Biol.

[B17] Locke JC, Kozma-Bognar L, Gould PD, Feher B, Kevei E, Nagy F, Turner MS, Hall A, Millar AJ (2006). Experimental validation of a predicted feedback loop in the multi-oscillator clock of Arabidopsis thaliana. Molecular Systems Biology.

[B18] Zeilinger MN, Farre EM, Taylor SR, Kay SA, Doyle FJ (2006). A novel computational model of the circadian clock in Arabidopsis that incorporates PRR7 and PRR9. Molecular Systems Biology.

[B19] Ding Z, Doyle MR, Amasino RM, Davis SJ (2007). A complex genetic interaction between Arabidopsis thaliana TOC1 and CCA1/LHY in driving the circadian clock and in output regulation. Genetics.

[B20] Kolmos E, Schoof H, Pluemer M, Davis SJ (2008). Structural insights into the function of the core-circadian factor TIMING OF CAB2 EXPRESSION 1 (TOC1). J Circadian Rhythms.

[B21] Hazen SP, Schultz TF, Pruneda-Paz JL, Borevitz JO, Ecker JR, Kay SA (2005). LUX ARRHYTHMO encodes a Myb domain protein essential for circadian rhythms. Proc Natl Acad Sci USA.

[B22] Kuno N, Moller SG, Shinomura T, Xu X, Chua NH, Furuya M (2003). The novel MYB protein EARLY-PHYTOCHROME-RESPONSIVE1 is a component of a slave circadian oscillator in Arabidopsis. Plant Cell.

[B23] Salathia N, Davis SJ, Lynn JR, Michaels SD, Amasino RM, Millar AJ (2006). FLOWERING LOCUS C-dependent and -independent regulation of the circadian clock by the autonomous and vernalization pathways. BMC Plant Biol.

[B24] Riechmann JL, Heard J, Martin G, Reuber L, Jiang C, Keddie J, Adam L, Pineda O, Ratcliffe OJ, Samaha RR (2000). Arabidopsis transcription factors: genome-wide comparative analysis among eukaryotes. Science.

[B25] Stracke R, Werber M, Weisshaar B (2001). The R2R3-MYB gene family in Arabidopsis thaliana. Curr Opin Plant Biol.

[B26] Jakoby M, Weisshaar B, Droge-Laser W, Vicente-Carbajosa J, Tiedemann J, Kroj T, Parcy F (2002). bZIP transcription factors in Arabidopsis. Trends Plant Sci.

[B27] Zimmermann IM, Heim MA, Weisshaar B, Uhrig JF (2004). Comprehensive identification of Arabidopsis thaliana MYB transcription factors interacting with R/B-like BHLH proteins. Plant J.

[B28] Edwards KD, Anderson PE, Hall A, Salathia NS, Locke JC, Lynn JR, Straume M, Smith JQ, Millar AJ (2006). FLOWERING LOCUS C mediates natural variation in the high-temperature response of the Arabidopsis circadian clock. Plant Cell.

[B29] Zimmermann P, Hirsch-Hoffmann M, Hennig L, Gruissem W (2004). GENEVESTIGATOR. Arabidopsis microarray database and analysis toolbox. Plant Physiol.

[B30] Straume M (2004). DNA microarray time series analysis: automated statistical assessment of circadian rhythms in gene expression patterning. Methods Enzymol.

[B31] Panda S, Hogenesch JB, Kay SA (2003). Circadian light input in plants, flies and mammals. Novartis Found Symp.

[B32] Edwards KD, Lynn JR, Gyula P, Nagy F, Millar AJ (2005). Natural allelic variation in the temperature-compensation mechanisms of the Arabidopsis thaliana circadian clock. Genetics.

[B33] Doyle MR, Davis SJ, Bastow RM, McWatters HG, Kozma-Bognar L, Nagy F, Millar AJ, Amasino RM (2002). The ELF4 gene controls circadian rhythms and flowering time in Arabidopsis thaliana. Nature.

[B34] Hanano S, Domagalska MA, Nagy F, Davis SJ (2006). Multiple phytohormones influence distinct parameters of the plant circadian clock. Genes Cells.

[B35] Salome PA, Mcclung CR (2005). What makes the Arabidopsis clock tick on time? A review on entrainment. Plant Cell and Environment.

[B36] Kolmos E, Davis SJ (2007). Rho-Related Signals in Time-Specific Light Perception. Current Biology.

[B37] Ueda HR, Hayashi S, Chen W, Sano M, Machida M, Shigeyoshi Y, Iino M, Hashimoto S (2005). System-level identification of transcriptional circuits underlying mammalian circadian clocks. Nat Genet.

[B38] Jakoby M, Wang HY, Reidt W, Weisshaar B, Bauer P (2004). FRU (BHLH029) is required for induction of iron mobilization genes in Arabidopsis thaliana. FEBS Lett.

[B39] Clough SJ, Bent AF (1998). Floral dip: a simplified method for Agrobacteriummediated transformation of Arabidopsis thaliana. Plant Journal.

[B40] Domagalska MA, Schomburg FM, Amasino RM, Vierstra RD, Nagy F, Davis SJ (2007). Attenuation of brassinosteroid signaling enhances FLC expression and delays flowering. Development.

[B41] Plautz JD, Straume M, Stanewsky R, Jamison CF, Brandes C, Dowse HB, Hall JC, Kay SA (1997). Quantitative analysis of Drosophila period gene ranscription in living animals. J Biol Rhythms.

[B42] Dowson-Day MJ, Millar AJ (1999). Circadian dysfunction causes aberrant hypocotyl elongation patterns in Arabidopsis. Plant J.

[B43] Thain SC, Hall A, Millar AJ (2000). Functional independence of circadian clocks that regulate plant gene expression. Curr Biol.

[B44] Hall A, Bastow RM, Davis SJ, Hanano S, McWatters HG, Hibberd V, Doyle MR, Sung S, Halliday KJ, Amasino RM (2003). The TIME FOR COFFEE gene maintains the amplitude and timing of Arabidopsis circadian clocks. Plant Cell.

