# Supplementary material for: A systematic survey in Arabidopsis thaliana of transcription factors that modulate circadian parameters
Source: BMC Genomics. 2008 Apr 21;9:182. doi: 10.1186/1471-2164-9-182 (PMC2410138; doi:10.1186/1471-2164-9-182)
Supplement: Additional file 1 — Supplemental Table 1 – Circadian regulated MYB, bHLH and bZIP genes. Circadian expression values available in the public database GENEVESTIGATOR were scored for circadian regulation using the modified cosinor analysis program COSOPT. Mean of expression levels, period length, phase values (ZT) and pMMC-β are represented. COSOPT (pMMC-β < 0.05) without linear regression are listed here. [file 1471-2164-9-182-S1.pdf]

Supplemental table 1.

Circadian-regulated MYB, bHLH and bZIP genes

GeneID	At number	Mean Exp.Lev.	Period (h)	Phase (ZT h)	pMMC-Beta
bHLH65/*PIL6*	At3g59060	1574.70	23.40	30.33	0.004
bHLH9/*PIF4*	At2g43010	1577.20	23.40	32.62	0.005
bHLH16	At4g00050	2330.60	23.50	47.93	0.005
bZIP3	At5g15830	222.75	24.00	31.28	0.007
bZIP54/*GBF2*	At4g01120	498.45	20.60	45.93	0.007
bHLH4/*AtMYC4*	At4g17880	662.77	23.20	48.65	0.008
MYB106	At3g01140	737.64	24.00	43.04	0.008
MYB28	At5g61420	2214.50	24.40	42.56	0.008
bZIP64	At5g06839	447.28	22.70	26.21	0.009
bHLH15/*PIL5*	At2g20180	680.73	26.60	26.88	0.009
MYB91	At2g37630	3688.00	24.00	42.08	0.011
bHLH153	At1g05710	480.92	24.90	45.35	0.011
bHLH26/*HFR1*	At1g02340	768.14	24.20	30.64	0.012
bZIP22/*TGA3*	At1g22070	1421.10	22.20	41.58	0.012
bZIP1	At5g49450	615.40	24.20	33.81	0.012
bHLH73/*ALCATRAZ*	At5g67110	426.42	23.20	47.53	0.012
bHLH147	At3g17100	1146.40	24.30	32.40	0.013
bHLH34	At3g23210	1199.70	27.10	41.13	0.014
bZIP37/*ABF3*	At4g34000	1230.60	23.10	27.78	0.016
bHLH64	At2g18300	1566.70	24.90	31.94	0.017
bHLH150	At3g05800	675.22	23.60	37.60	0.019
MYB32	At4g34990	538.59	25.30	41.20	0.019
bHLH136	At5g39860	625.80	24.80	29.33	0.020
bZIP58	At1g13600	180.43	26.20	27.72	0.021
bHLH142	At5g64340	928.39	26.30	36.38	0.021
bZIP63/*BZO3H3*	At5g28770	559.26	23.60	31.34	0.023
MYB44	At5g67300	1159.50	24.60	27.26	0.026
bHLH130	At2g42280	326.71	20.80	46.39	0.027
bHLH104	At4g14410	1218.90	21.50	47.69	0.028
MYB3	At1g22640	1818.40	25.70	27.93	0.031
bHLH148	At3g06590	963.85	22.30	42.96	0.032
MYB59	At5g59780	1779.60	24.70	31.85	0.032
bZIP47/*TGA1*	At5g65210	1435.30	27.30	27.24	0.032
bZIP61	At3g58120	405.24	24.10	33.50	0.033
MYB48	At3g46130	445.75	25.20	35.79	0.035
bHLH7	At1g03040	1019.70	25.10	39.24	0.037
bHLH128	At1g05805	638.07	23.70	35.83	0.038
bZIP11/*ATB2*	At4g34590	1815.70	20.70	49.11	0.039
bZIP41/*GBF1*	At4g36730	1414.00	22.10	34.75	0.039
bZIP56/*HY5*	At5g11260	524.63	22.30	49.17	0.040
MYB75/*PAP1*	At1g56650	771.68	26.10	43.47	0.045
bZIP9/*CPRF-2*	At5g24800	1463.20	22.40	36.24	0.047
