# Supplementary material for: A systematic survey in Arabidopsis thaliana of transcription factors that modulate circadian parameters
Source: BMC Genomics. 2008 Apr 21;9:182. doi: 10.1186/1471-2164-9-182 (PMC2410138; doi:10.1186/1471-2164-9-182)
Supplement: Additional file 2 — Supplemental Table 2 – Estimated period length of transgenic lines overexpressing MYB transcription factors. Mean circadian periods of leaf movement in Arabidopsis plants misexpressing transcription factors and control seedlings, estimated with BRASS. S.E.M.: standard error of the mean, n: number of contributing leaf traces. [file 1471-2164-9-182-S2.pdf]

Supplement table 2.

Estimated period length of transgenic lines over-expressing MYB transcription factors

genes	At number	lines	n	Period	±	SE	comments	control plants n	control plants Period	±	SE
MYB5	**At3g13540**	***A***	29	24.35	±	0.37		29	24.39	±	0.28
		B	11	24.19	±	0.13		12	23.87	±	0.16
MYB6	**At4g38620**	***A***	22	24.19	±	0.37		22	24.73	±	0.21
		B	12	24.77	±	0.25		10	24.72	±	0.22
MYB55	**At4g01680**	***C***	32	24.38	±	0.21		25	24.41	±	0.20
		D	26	24.60	±	0.25		18	24.04	±	0.21
		K	13	24.56	±	0.14		12	24.19	±	0.31
MYB64	**At5g11050**	***A***	16	24.04	±	0.36		18	24.33	±	0.25
		B	28	24.67	±	0.39		31	24.11	±	0.35
		C	11	24.64	±	0.66		21	24.61	±	0.37
		D	11	23.70	±	0.23		9	24.02	±	0.22
MYB95	**At1g74430**	***A***	12	23.78	±	0.33		9	24.02	±	0.22
		B	24	24.50	±	0.57		20	24.26	±	0.17
		F	8	24.42	±	0.28		11	25.55	±	0.57
		G	12	24.46	±	0.16		10	25.07	±	0.56
MYB98	**At4g18770**	***E***	31	24.24	±	0.34		28	24.56	±	0.31
		G	34	25.17	±	0.49		33	24.07	±	0.25
MYB103	**At1g63910**	***B***	37	24.23	±	0.37		37	23.91	±	0.36
		C	16	24.57	±	0.19		21	24.30	±	0.28
		E	24	24.49	±	0.34		21	24.30	±	0.28
		F	15	24.50	±	0.28		17	24.80	±	0.28
MYB107	**At3g02940**	***I***	16	24.67	±	0.63		17	24.80	±	0.28
MYB111	**At5g49330**	***A***	44	24.64	±	0.30		37	24.35	±	0.29
		D	33	24.87	±	0.31		38	24.27	±	0.32
		E	14	24.68	±	0.60		11	23.24	±	0.50
MYB114	**At1g66380**	***A***	25	24.44	±	0.61		42	24.30	±	0.30
		B	15	24.69	±	0.33		16	24.70	±	0.60
MYB116/CPM7	**At1g25340**	***E***	23	24.52	±	0.30		20	24.55	±	0.41
		F	19	24.65	±	0.19		14	24.26	±	0.35
MYB124	**At1g14350**	***1***	11	24.96	±	0.34		4	25.05	±	0.36
		2	14	24.64	±	0.24		11	24.24	±	0.11
MYB84	**At3g49690**	***1***	23	24.55	±	0.23		15	24.64	±	0.24
		3	16	23.77	±	0.32		13	23.94	±	0.23
MYB91	**At2g37630**	***1***	11	24.44	±	0.33		11	24.13	±	0.30
		2	3	24.20	±	0.29		6	24.43	±	0.40
		5	15	25.10	±	0.77		6	24.25	±	0.32
		7	12	26.32	±	1.13	long*	9	24.03	±	0.29
MYB79	**At4g13480**	***1***	42	24.70	±	0.26		38	24.29	±	0.30
		2	39	25.04	±	0.49		30	24.16	±	0.27
		3	23	25.21	±	0.34	long*	19	23.83	±	0.19
		4	23	24.44	±	0.22		20	24.07	±	0.20
MYB115	**At5g40360**	***3***	36	24.69	±	0.39		42	24.34	±	0.17
		5	34	25.34	±	0.37		44	24.77	±	0.25
		7	19	25.00	±	0.19		23	24.74	±	0.35
		8	25	25.86	±	0.39	long*	13	24.84	±	0.20
MYB29	**At5g07690**	***2***	52	24.60	±	0.25		36	24.61	±	0.24
		3	39	24.12	±	0.34		31	24.52	±	0.26
MYB101	**At2g32460**	***6***	39	24.56	±	0.23		28	24.52	±	0.25
		7	31	25.70	±	0.29	long*	23	24.61	±	0.32
MYB109	**At3g55730**	***1***	33	24.65	±	0.40		25	24.48	±	0.34
		3	23	24.45	±	0.24		29	24.31	±	0.21
		4	27	24.77	±	0.32		16	24.12	±	0.25
		5	9	24.11	±	0.13		10	24.32	±	0.23
MYB51	**At1g18570**	***1***	19	24.07	±	0.24		17	24.20	±	0.28
		4	22	24.45	±	0.35		15	24.66	±	0.25
		7	13	24.44	±	0.25		11	24.80	±	0.32
MYB43	**At5g16660**	***1***	38	24.62	±	0.38		25	24.72	±	0.25
		2	29	25.04	±	0.40		24	24.69	±	0.46

continued in next page

MYB122	**At1g74080**	***1***	12	24.60	±	0.32		17	24.72	±	0.53
		3	42	24.44	±	0.29		28	24.27	±	0.15
MYB85	**At4g22680**	***1***	36	24.46	±	0.22	long*	27	24.01	±	0.15
		2	35	24.54	±	0.37		33	24.04	±	0.30
		3	11	24.17	±	0.39		9	24.99	±	0.25
		4	21	25.40	±	0.59	long*	13	24.67	±	0.49
MYB99	**At5g62320**	***2***	15	24.77	±	0.16		13	24.67	±	0.49
		5	19	25.21	±	0.12		16	24.87	±	0.24
MYB3R2	**At4g00540**	***1***	43	24.44	±	0.46	phase	31	24.96	±	0.27
		2	28	24.53	±	0.29	phase	26	24.60	±	0.30
		5	12	24.00	±	0.19	phase	9	23.73	±	0.21
		6	11	26.69	±	0.28	phase/long*	6	25.57	±	0.28
		7	12	25.06	±	0.33	phase	12	24.39	±	0.29
MYB113	**At1g66370**	***2***	17	24.71	±	0.31		18	24.98	±	0.28
		3	28	24.60	±	0.31		15	24.91	±	0.22
MYB9	**At5g16770**	***1***	41	24.50	±	0.15		25	24.70	±	0.23
		2	38	24.69	±	0.13		29	24.42	±	0.22
		3	25	24.39	±	0.19		19	24.28	±	0.18
		4	20	24.87	±	0.26		17	24.15	±	0.12
MYB30/HSR1	**At3g28910**	***2***	32	25.41	±	0.41		29	24.30	±	0.17
		3	20	24.39	±	0.21		18	24.31	±	0.17
		4	5	25.05	±	0.22	long*	8	24.63	±	0.14
		5	6	24.98	±	0.13	long*	9	24.17	±	0.20
MYB59	**At5g59780**	***2***	30	24.76	±	0.34	long*	26	24.06	±	0.22
		13	17	24.57	±	0.28		25	24.33	±	0.20
		17	5	24.30	±	0.18		8	23.57	±	0.20
MYB62	**At1g68320**	***7***	15	24.30	±	0.55		13	24.30	±	0.14
		8	23	24.19	±	0.37		16	24.64	±	0.14
		16	12	24.75	±	0.24		9	23.86	±	0.15
MYB102/M4	**At4g21440**	***1***	42	24.09	±	0.24		33	24.23	±	0.18
		2	21	24.57	±	0.23		18	25.34	±	0.24
		4	13	25.27	±	0.19		7	24.60	±	0.44
MYB78	**At5g49620**	***9***	28	24.98	±	0.30		20	25.00	±	0.35
		10	21	24.58	±	0.29		20	24.70	±	0.32
MYB94	**At3g47600**	***1***	17	24.81	±	0.14		12	24.37	±	0.22
		2	20	24.98	±	0.32		18	24.58	±	0.30
MYB3R3a	**At3g09370**	***A***	28	24.86	±	0.26		28	24.08	±	0.32
		B	32	25.83	±	0.31	long*	32	23.81	±	0.23
		D	15	24.53	±	0.29		18	22.98	±	0.31
		F	32	24.58	±	0.62		29	24.59	±	0.19
MYB3R3b	**At3g09370**	***K***	37	24.75	±	0.21		31	24.55	±	0.22
		L	34	24.79	±	0.23		35	24.92	±	0.20
MYB3R4	**At5g11510**	***C***	36	25.51	±	0.35		32	24.94	±	0.31
		K	26	25.07	±	0.20		30	24.87	±	0.43
MYB4R1	**At3g18100**	***F***	12	24.70	±	0.15		9	24.37	±	0.23
MYB26	**At3g13890**	***I***	35	25.06	±	0.24		30	24.94	±	0.31
		L	6	25.07	±	0.28		21	24.18	±	0.20
		H	5	24.23	±	0.19		12	25.20	±	0.17
MYB3R5	**At5g02320**	***1***	15	25.40	±	0.30		5	23.81	±	0.55
		3	13	24.63	±	0.03		7	24.14	±	0.32
