# Supplementary material for: A systematic survey in Arabidopsis thaliana of transcription factors that modulate circadian parameters
Source: BMC Genomics. 2008 Apr 21;9:182. doi: 10.1186/1471-2164-9-182 (PMC2410138; doi:10.1186/1471-2164-9-182)
Supplement: Additional file 3 — Supplemental Table 3 – Estimated period length of transgenic lines overexpressing bHLH transcription factors. Mean circadian periods of leaf movement in Arabidopsis plants misexpressing transcription factors and control seedlings, estimated with BRASS. S.E.M.: standard error of the mean, n: number of contributing leaf traces. [file 1471-2164-9-182-S3.pdf]

Supplement table 3.

Estimated period length of transgenic lines over-expressing bHLH transcription factors

								control plants			
**genes**	**At number**	**line**	**n**	**Period**	**±**	**SE**	**comments**	**n**	**Period**	**±**	**SE**
bHLH1/GL3	**At5g41315**	***2***	23	24.91	±	0.20		18	24.72	±	0.15
		3	21	24.79	±	0.26		22	24.71	±	0.10
bHLH3	**At4g16430**	***5***	56	24.83	±	0.23		51	24.65	±	0.23
		7	31	25.02	±	0.21		31	24.36	±	0.31
		10	31	24.48	±	0.25		29	25.03	±	0.19
bHLH7	**At1g03040**	***1***	21	25.30	±	0.31		18	25.21	±	0.08
		5	14	25.02	±	0.17		15	25.63	±	0.16
bHLH12/AtMYC1	**At4g00480**	***1***	18	25.17	±	0.27		18	25.38	±	0.23
		2	23	25.02	±	0.20		18	25.42	±	0.37
bHLH16	**At4g00050**	***1***	32	25.04	±	0.25		29	25.39	±	0.36
		4	28	24.91	±	0.20		29	24.45	±	0.41
		5	12	24.82	±	0.41		14	24.95	±	0.40
		6	8	25.00	±	0.43		10	24.84	±	0.12
bHLH19	**At2g22760**	***1***	7	24.82	±	0.22		10	24.84	±	0.12
		3	14	24.47	±	0.64		11	25.07	±	0.17
bHLH29	**At2g28160**	***1.1***	36	24.69	±	0.20		30	24.54	±	0.15
		1.2	31	24.80	±	0.16		27	24.81	±	0.28
bHLH101	**At5g04150**	***3***	12	24.64	±	0.23		11	24.44	±	0.33
bHLH7	**At1g03040**	***E***	21	24.51	±	0.54		16	24.31	±	0.35
		H	28	24.25	±	0.33		29	24.33	±	0.42
		J	13	26.31	±	0.99		11	24.63	±	0.11
bHLH5/ATR2/AtMYC1	**At5g46760**	***B***	36	24.65	±	0.29		28	24.40	±	0.27
		C	23	24.37	±	0.25		30	24.18	±	0.39
		E	12	30.02	±	3.88		11	24.77	±	0.26
bHLH14	**At4g00870**	***C***	28	24.74	±	0.25		30	24.57	±	0.26
		D	44	25.22	±	0.29	long*	41	24.73	±	0.27
		J	35	25.56	±	0.35	long*	35	23.86	±	0.27
		K	32	24.98	±	0.43		31	24.67	±	0.26
bHLH18	**At2g22750**	***A***	23	24.82	±	0.31		25	24.14	±	0.32
		B	28	24.98	±	0.30		25	23.89	±	0.27
		C	13	24.62	±	0.16		15	24.18	±	0.37
bHLH28	**At5g46830**	***A***	12	24.54	±	0.28		12	23.97	±	0.10
		B	18	23.97	±	0.19		18	23.78	±	0.22
bHLH38/ORG2	**At3g56970**	***B***	15	23.88	±	0.44		18	25.79	±	0.26
		C	18	25.56	±	0.21		22	25.41	±	0.23
bHLH39/ORG3	**At3g56980**	***E***	35	25.26	±	0.34	long*	49	24.49	±	0.29
		G	24	24.37	±	0.29		28	24.58	±	0.30
		I	27	24.73	±	0.69		33	24.20	±	0.20
		J	5	24.53	±	0.30		12	24.27	±	0.24
		K	9	24.64	±	0.14		9	24.46	±	0.29
bHLH42/TT8	**At4g09820**	***C***	11	24.54	±	0.53		21	24.36	±	0.26
		E	10	24.58	±	0.16		9	24.37	±	0.09
		J	7	24.06	±	0.17		4	25.87	±	0.50
		L	15	27.57	±	0.86		18	24.90	±	0.42
bHLH50/BBE3	**At1g73830**	***A***	14	25.19	±	0.44		18	24.90	±	0.42
		E	39	25.11	±	0.62		30	24.68	±	0.20
bHLH68	**At4g29100**	***A***	17	24.91	±	0.18	long*	27	24.64	±	0.31
		B	32	25.14	±	0.24		31	24.85	±	0.20
bHLH69	**At4g30980**	***A***	47	25.20	±	0.44	phase	42	24.98	±	0.18
		B	51	25.39	±	0.33	phase/long*	51	24.61	±	0.21
		D	17	24.92	±	0.21	phase	18	24.28	±	0.15
		E	8	24.70	±	0.37	phase	4	25.87	±	0.50
		I	22	24.19	±	0.25	phase	21	24.95	±	0.32
bHLH72	**At5g61270**	***A***	27	24.93	±	0.34		33	24.76	±	0.33
		B	24	25.92	±	0.40	long*	22	25.00	±	0.30
		C	5	24.42	±	0.35		10	24.83	±	0.42
		D	10	24.37	±	0.22		9	24.63	±	0.17

continued in next page

bHLH81	**At4g09180**	***B***	24	25.33	±	0.90		19	24.44	±	0.35
		C	17	24.52	±	0.41		17	24.52	±	0.23
bHLH87	**At3g21330**	***B***	20	24.33	±	0.32		17	24.52	±	0.23
		C	17	24.87	±	0.21		18	24.56	±	0.52
bHLH88	**At5g67060**	***D***	22	24.65	±	0.28		18	24.19	±	0.31
		E	11	24.83	±	0.27		20	24.98	±	0.39
		N	8	24.82	±	0.15		6	25.37	±	1.12
bHLH91	**At2g31210**	***F***	5	25.25	±	0.19		10	24.83	±	0.42
		L	13	24.57	±	0.08		9	24.86	±	0.20
bHLH92	**At5g43650**	***A***	39	25.17	±	0.33	long	11	23.96	±	0.26
		B	20	24.85	±	0.28	long	9	24.31	±	0.23
		D	31	24.90	±	0.34	long	11	24.40	±	0.24
		E	36	24.69	±	0.37	long	11	23.91	±	0.20
bHLH100	**At2g41240**	***D***	9	25.23	±	0.74		6	24.20	±	0.26
		E	20	24.12	±	0.24		10	24.17	±	0.24
bHLH105	**At5g54680**	***E***	20	24.05	±	0.26		19	24.03	±	0.18
		I	17	24.59	±	0.53		16	24.19	±	0.21
bHLH125	**At1g62975**	***B***	30	24.55	±	0.35		23	24.01	±	0.32
		C	41	24.78	±	0.29		39	24.02	±	0.27
		D	21	25.02	±	0.45		27	24.51	±	0.49
		H	6	24.43	±	0.27		10	24.06	±	0.37
bHLH6/RAP1/AtMYC2	**At1g32640**	***A***	13	24.87	±	0.41		10	23.96	±	0.30
		B	27	24.52	±	0.26		32	24.58	±	0.21
		C	29	24.91	±	0.20	long*	32	23.91	±	0.20
		D	9	24.90	±	0.28	long*	10	24.46	±	0.34
