# Supplementary material for: A systematic survey in Arabidopsis thaliana of transcription factors that modulate circadian parameters
Source: BMC Genomics. 2008 Apr 21;9:182. doi: 10.1186/1471-2164-9-182 (PMC2410138; doi:10.1186/1471-2164-9-182)
Supplement: Additional file 4 — Supplemental Table 4 – Estimated period length of transgenic lines overexpressing bZIP transcription factors. Mean circadian periods of leaf movement in Arabidopsis plants misexpressing transcription factors and control seedlings, estimated with BRASS. S.E.M.: standard error of the mean, n: number of contributing leaf traces. [file 1471-2164-9-182-S4.pdf]

Supplement table 4.

Estimated period length of transgenic lines over-expressing bZIP transcription factors

genes	At number	line	n	Period	±	SE	comments	control plants n	Period	±	SE
bZIP48	**At2g04038**	***C***	6	23.62	±	0.49	high RAE	17	25.01	±	0.66
		G	6	25.00	±	0.78		22	24.49	±	0.31
		J	8	24.74	±	1.19		7	24.54	±	0.58
bZIP50	**At1g77920**	***B***	11	24.79	±	0.37		12	23.87	±	0.16
		D	39	24.04	±	0.19		30	23.95	±	0.30
		E	6	24.63	±	0.24		11	24.66	±	0.20
bZIP51/VIP1	**At1g43700**	***E***	17	24.63	±	0.31		10	23.89	±	0.15
bZIP58	**At1g13600**	***C***	13	24.61	±	0.37		10	23.96	±	0.30
