# Supplementary figures and images for: A systematic survey in Arabidopsis thaliana of transcription factors that modulate circadian parameters
Source: BMC Genomics. 2008 Apr 21;9:182. doi: 10.1186/1471-2164-9-182 (PMC2410138; doi:10.1186/1471-2164-9-182)

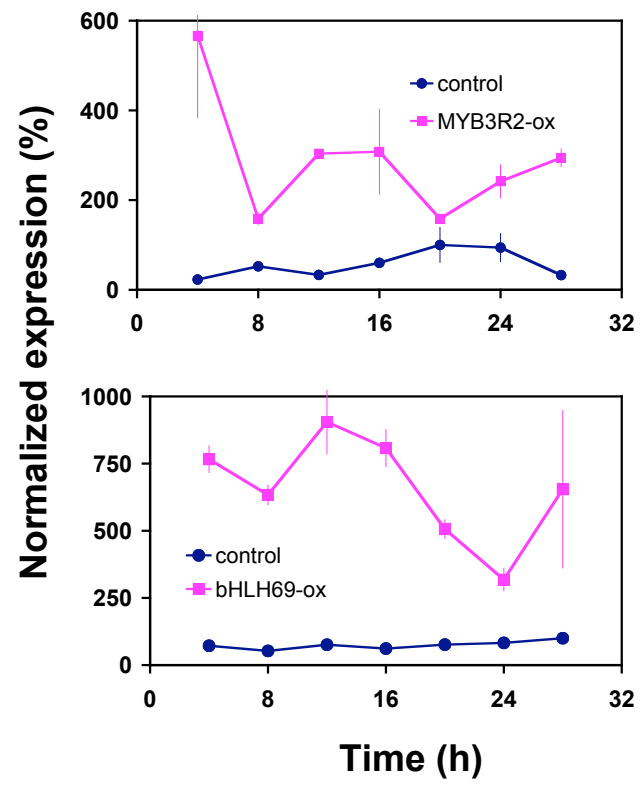

Figure S1

Supplement: Additional file 5 — Figure S1 – Confirmation of over-expression of MYB3R2 and bHLH69. Replicate seedlings from wild-type plants maintained under constant light were harvested every 4 hours. Total RNA was the substrate for RT-PCR of the coding regions of the transcription factors MYB3R2, bHLH69 and bHLH92. Results are presented as proportional to the average value after normalization with respect to TUB. (A) MYB3R2 and (B) bHLH69. [file 1471-2164-9-182-S5.pdf]

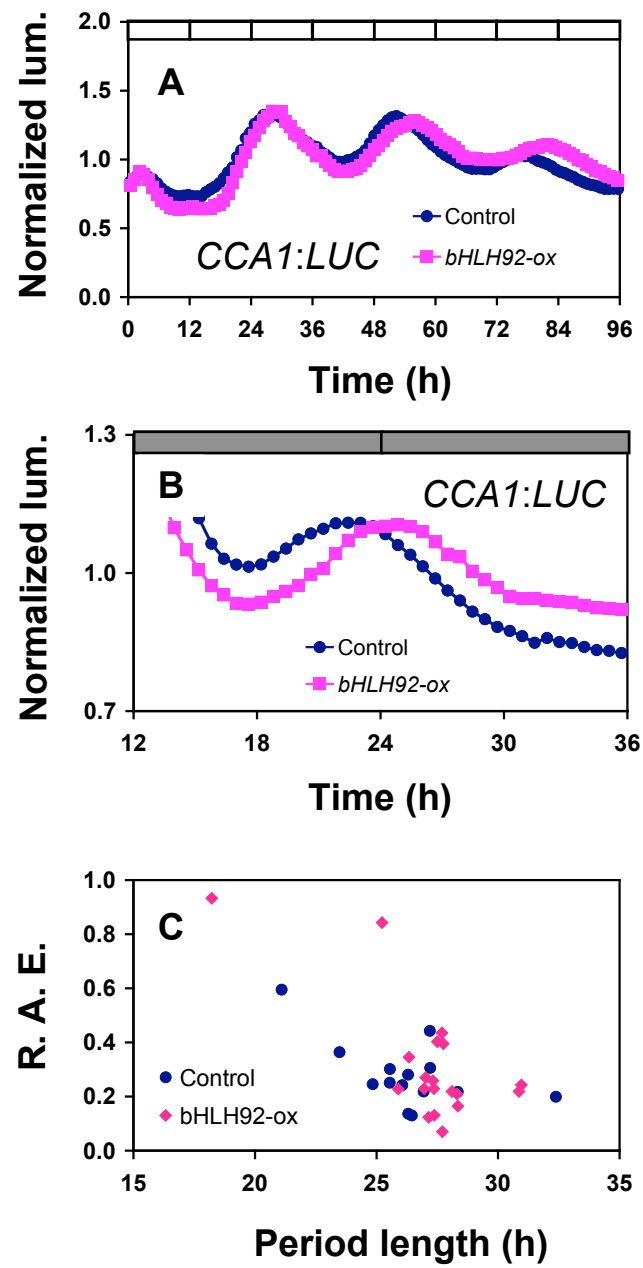

Figure S2

Supplement: Additional file 6 — Figure S2 – Confirmation of transcriptional clock phenotype of bHLH92-ox in constant light and in constant dark. Seedlings harboring CCA1:LUC reporter genes were monitored for 4–5 days both under LL (A) or in DD (B). Representative traces of rhythmic expression of ox-plants (pink squares) and wild-type (blue circles) are shown. (C) Relative Amplitude Error (R. A. E.) calculated from the data under LL was plotted against period (h). bHLH92-ox exhibited a slightly long periodicity phenotype. [file 1471-2164-9-182-S6.pdf]

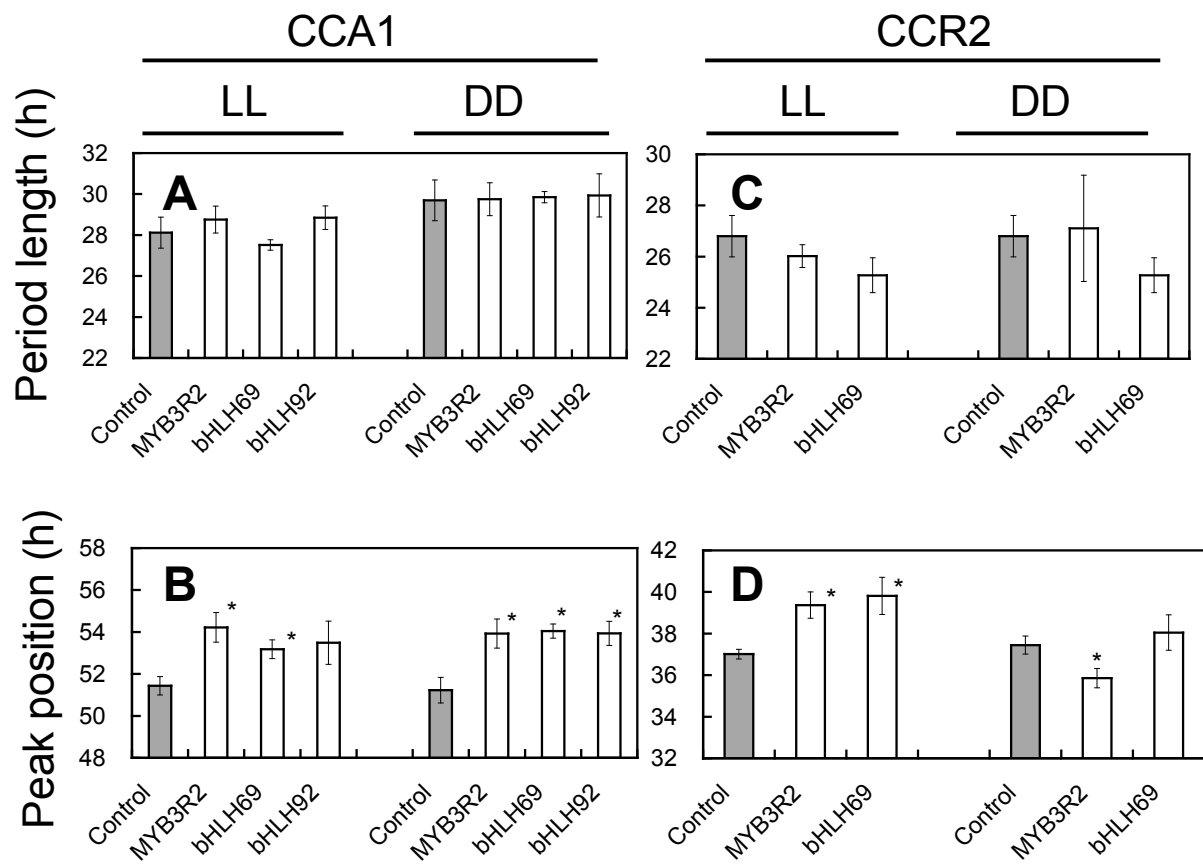

\*  $P < 0.01$

Figure S3

Supplement: Additional file 7 — Figure S3 – Estimated period and phase of MYB3R2-ox, bHLH69-ox and bHLH92-ox. Estimated period length and phase values were calculated by BRASS. (A) Estimated period of CCA1 rhythm. (B) Peak positions of second peak in CCA1 rhythm. (C) Estimated period of CCR2 rhythm. (D) Peak positions of second peak in CCR2 rhythm. Data are presented as mean ± S.E. with n of 12–24 plants. * P = 0.01. No significant difference in periodicity was observed (A and C). [file 1471-2164-9-182-S7.pdf]

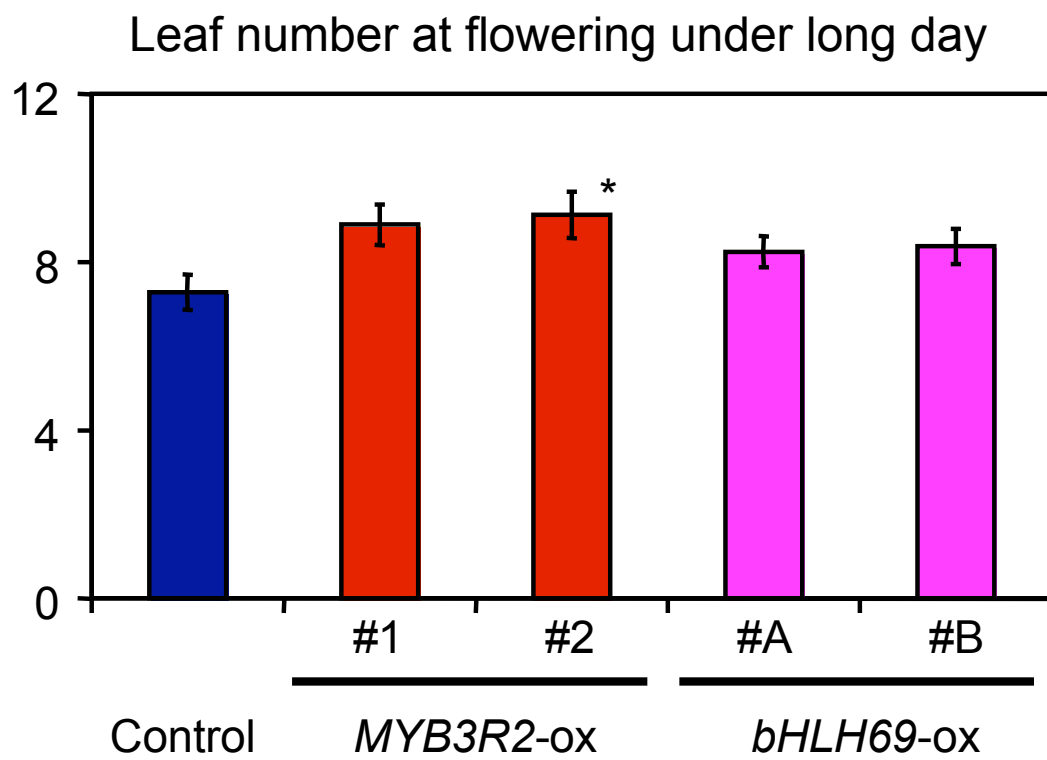

Figure S4

\*  $P = 0.038$

Supplement: Additional file 8 — Figure S4 – The effects of over-expression of MYB3R2 and bHLH69. Flowering time of MYB3R2-ox and bHLH69-ox plants was measured under long day. Leaf number at flowering time were plotted against the genotype and line tested. Data are presented as mean ± S.E. with n of 9–14 plants. * P = 0.038. No significant differences were detected in the flowering time of other lines. [file 1471-2164-9-182-S8.pdf]
